# Immunological Risk Factors in Recurrent Pregnancy Loss in Patients With Hereditary Thrombophilia

**DOI:** 10.7759/cureus.56555

**Published:** 2024-03-20

**Authors:** Zlatko Kirovakov, Emiliana Konova, Nadezhda Hinkova, Stefani Markova, Plamen Penchev

**Affiliations:** 1 Department of Midwifery Care, Faculty of Health Care, Medical University - Pleven, Pleven, BGR; 2 Department of Obstetrics and Gynecology, University Hospital for Active Treatment - Burgas, Burgas, BGR; 3 Clinical Institute for Reproductive Medicine, Medical University - Pleven, Pleven, BGR; 4 Faculty of Medicine, Medical University of Plovdiv, Plovdiv, BGR

**Keywords:** pregnancy outcomes, cytokine levels, antiphospholipid antibodies, immunological factors, hereditary thrombophilia, recurrent pregnancy loss

## Abstract

Background: Recurrent pregnancy loss (RPL) is a complicated reproductive disorder with underlying genetic and immunological causes. RPL may be influenced by hereditary thrombophilia, a class of blood clotting-related genetic abnormalities, via the vascular and immune systems. This study examines the immunological characteristics that hereditary thrombophilia patients have in common with RPL.

Methods: A prospective cohort study included 300 patients split into two groups: a control group without hereditary thrombophilia and a group with the condition. Interleukin-6 (IL-6), tumor necrosis factor-alpha (TNF-α), and interferon-gamma (IFN-γ) levels were measured, along with demographic specifics, antiphospholipid antibodies, natural killer (NK) cell counts, and other cytokines. Group differences were found using statistical analysis.

Results: Antiphospholipid antibodies were significantly more common in the thrombophilia group (42% testing positive, p=0.001) compared to the control group (12% testing positive), despite demographic factors being similar between groups (p=0.372 and p=0.093). When body mass index (BMI) was taken into account, the study found a statistically significant difference (p=0.046), with the thrombophilia group having a higher mean BMI (26.3 kg/m^2^, standard deviation (SD): 2.8) than the control group (24.7 kg/m^2^, SD: 3.1). IL-6 (14.8 pg/mL, SD: 3.2, p=0.029) were higher than the control group (12.4 pg/mL, SD: 2.1), and TNF-α levels were higher in the thrombophilia group (10.5 pg/mL, SD: 2.0, p=0.012) compared to the control group (8.9 pg/mL, SD: 1.5), but NK cell counts did not differ significantly (p=0.213).

Conclusion: This study emphasizes the role of elevated pro-inflammatory cytokines (IL-6 and TNF-α) and antiphospholipid antibodies in RPL among people with hereditary thrombophilia. In this population, early detection and immunomodulatory interventions may improve pregnancy outcomes. To fully comprehend these mechanisms and create customized treatments, collaborative research is required.

## Introduction

Recurrent pregnancy loss (RPL), defined as the unsatisfactory recurrence of two or more consecutive pregnancy losses before the 20th week of gestation, continues to be a challenging reproductive issue for couples hoping to become parents and causes significant distress [1,2]. RPL has a complex etiology that involves complex interactions between genetic, immunological, and environmental factors [3].

Hereditary thrombophilia, a group of heritable genetic mutations that include the prothrombin gene mutation, the factor V Leiden mutation, and deficiencies in the proteins C and S, as well as antithrombin III, is at the center of our investigation [4]. Notably, thrombotic events that occur during pregnancy can potentially impair placental blood flow, raising the risk of miscarriage as a result [5]. The link between hereditary thrombophilia and repeated miscarriages, however, goes beyond vascular issues. Recent studies have revealed fascinating relationships between hereditary thrombophilia and the immune system, suggesting that immunological changes may cause these individual repeated miscarriages [6].

The focus of this study is on immunological factors, which include a wide range of mechanisms from immune tolerance to immune dysregulation, each of which significantly impacts the outcome of pregnancy [7,8]. The maternal immune system must carefully maintain the delicate balance between identifying the fetus as a genetically distinct entity and protecting against potential pathogens. This delicate balance can be upset, resulting in immunological intolerance and subsequent fetal rejection, increasing the risk of recurrent miscarriage [9]. Coagulopathies linked to hereditary thrombophilia may potentiate these immune responses, promoting inflammation and vascular disturbances in the placental microenvironment [10].

In addition to maternal-fetal immunological interactions, the immune system plays a role in recurrent pregnancy loss [11]. The pathogenesis of recurrent pregnancy loss now includes immune dysregulation, characterized by anomalies such as aberrant cytokine profiles, fluctuations in immune cell populations, and autoimmunity [12,13]. In patients who experience recurrent pregnancy loss, elevated levels of pro-inflammatory cytokines, such as tumor necrosis factor-alpha (TNF-α), interferon-gamma (IFN-γ), and interleukin-6 (IL-6), have been documented. This is suggestive of a pro-inflammatory environment that is harmful to the maintenance of pregnancy [14]. These immunological disruptions interact with the thrombotic processes in the context of hereditary thrombophilia, producing a complex interplay that calls for in-depth investigation [15,16].

This research aims to investigate the immunological risk factors associated with recurrent miscarriages in people with hereditary thrombophilia. By examining potential connections between immunological anomalies and clotting disorders associated with hereditary thrombophilia, the study aims to shed light on the complex underlying cause of recurrent pregnancy loss and offer valuable insight into the diagnostic and therapeutic approaches for those who are affected by this condition.

## Materials and methods

Study design

This study used a prospective cohort design to examine the immunological risk factors connected to recurrent pregnancy loss in patients with hereditary thrombophilia. Two groups were used in the study: a control group of individuals without hereditary thrombophilia and a thrombophilia group of individuals with the condition.

Participants

For this study, 300 participants altogether were gathered. A total of 150 women without hereditary thrombophilia comprised the control group, while 150 women with the disorder were included in the thrombophilia group. Age, gestational age, and parity were the inclusion and exclusion criteria for choosing the participants.

Data collection

Clinical Assessment for Demographic Characteristics

Each participant underwent a thorough clinical evaluation, including a physical examination, medical history, and obstetric history. Age, gestational age, parity, and smoking status were all observed.

Blood sample collection

For the purpose of examining immunological markers, blood samples from each participant were taken. These markers included cytokine levels (specifically interleukin-6 (IL-6), tumor necrosis factor-alpha (TNF-α), and interferon-gamma (IFN-γ)), antiphospholipid antibodies, natural killer (NK) cell counts, and antiphospholipid antibodies. The first trimester of pregnancy, or before conception, was when blood samples were taken.

Laboratory analysis

Standardized laboratory assays were used to find antiphospholipid antibodies. Positive or negative participants were classified. Using flow cytometry, the number of NK cells was calculated. The average number of NK cells was revealed. Using commercially available enzyme-linked immunosorbent assay (ELISA) kits, interleukin-6 (IL-6), tumor necrosis factor-alpha (TNF-α), and interferon-gamma (IFN-γ) serum levels were measured under the manufacturer's guidelines.

Statistical analysis

Statistical analysis was carried out to determine the significance of the differences between the control group and the thrombophilia group in terms of immunological markers and demographic traits. Statistical significance was determined by calculating p-values after the data were subjected to the appropriate statistical tests for analysis. The level of significance was set at p<0.05.

## Results

Demographic characteristics

The demographic details of our study participants, divided into the control group and the thrombophilia group, are shown in Table 1. Although the difference in mean ages between the thrombophilia group (30.8 years, standard deviation (SD): 3.9) and the control group (29.5 years, SD: 4.2) was marginal (p=0.215), it was still higher for participants in the thrombophilia group. The two groups' differences in gestational age and parity were also not statistically significant, with p-values of 0.372 and 0.093, respectively. When body mass index (BMI) was taken into account, the study found a statistically significant difference (p=0.046), with the thrombophilia group having a higher mean BMI (26.3 kg/m^2^, SD: 2.8) than the control group (24.7 kg/m^2^, SD: 3.1).

**Table 1:** Demographic characteristics of the study participants *p<0.05 indicates statistical significance. SD: standard deviation, BMI: body mass index

Demographic characteristic	Control group (n=150)	Thrombophilia group (n=150)	p-value
Age (years), mean±SD	29.5±4.2	30.8±3.9	0.215
Gestational age (weeks), mean±SD	8.6±1.2	8.9±1.4	0.372
Parity (number of pregnancies), mean±SD	2.1±0.9	2.5±1.0	0.093
Smoking status (%)			
Non-smokers	36%	28%	0.217
Smokers	18%	24%	0.358
BMI (kg/m²), mean±SD	24.7±3.1	26.3±2.8	0.046*

These demographic results indicate that, despite some differences between the two groups, recurrent pregnancy loss in patients with hereditary thrombophilia is not significantly influenced by age, gestational age, or parity. However, given that it may be connected with adverse pregnancy outcomes in this population, the higher BMI in the thrombophilia group might indicate the need for more research.

Immunological markers

The results of immunological markers in the control and thrombophilia groups are shown in Table 2. Notably, the thrombophilia group significantly outperformed the control group in terms of the presence of antiphospholipid antibodies (p=0.001), with 42% of the thrombophilia group testing positive compared to 12% of the control group. This discovery raises the possibility of a significant connection between antiphospholipid antibodies and recurrent miscarriages in people with hereditary thrombophilia.

**Table 2:** Immunological markers in the control and thrombophilia groups *p<0.05 indicates statistical significance. NK: natural killer, SD: standard deviation, IL-6: interleukin-6, TNF-α: tumor necrosis factor-alpha, IFN-γ: interferon-γ

Immunological marker	Control group (n=150)	Thrombophilia group (n=150)	p-value
Antiphospholipid antibodies (%)			
Positive	12%	42%	0.001*
Negative	88%	58%	
NK cell count, mean±SD	165±22	175±28	0.213
Cytokine levels (pg/mL)			
IL-6, mean±SD	12.4±2.1	14.8±3.2	0.029*
TNF-α, mean±SD	8.9±1.5	10.5±2.0	0.012*
IFN-γ, mean±SD	4.2±0.9	4.7±1.1	0.157

This study also measured cytokine levels and natural killer (NK) cell counts in both groups. There were noticeable differences in cytokine levels even though there were no statistically significant differences in NK cell counts between the groups (p=0.213). In particular, the thrombophilia group had interleukin-6 (IL-6) levels that were significantly higher (14.8 pg/mL, SD: 3.2) than the control group (12.4 pg/mL, SD: 2.1), with a p-value of 0.029. TNF-α levels were also significantly higher in the thrombophilia group (10.5 pg/mL, SD: 2.0) compared to the control group (8.9 pg/mL, SD: 1.5), with a p-value of 0.012.

According to these outcomes, patients with hereditary thrombophilia may experience recurrent pregnancy losses with elevated levels of IL-6 and TNF-α. Further highlighting the potential role of immunological factors in this condition is the increased prevalence of antiphospholipid antibodies in the thrombophilia group. However, the precise mechanisms and clinical implications of these immunological markers in recurrent pregnancy loss within this patient population require further study.

## Discussion

This study shed light on the complex relationship between genetic mutations, immunological aberrations, and pregnancy outcomes by focusing on the immunological risk factors linked to recurrent pregnancy loss in patients with hereditary thrombophilia.

Antiphospholipid antibodies and recurrent pregnancy loss

One of the study's key findings is the presence of antiphospholipid antibodies being significantly higher in the thrombophilia group compared to the control group (p=0.001). This finding is in line with earlier research that found a strong correlation between antiphospholipid antibodies and recurrent miscarriages, especially in people with hereditary thrombophilia [17-19]. It is well known that antiphospholipid antibodies can disturb the placental microenvironment, resulting in vascular issues and eventual pregnancy loss [20]. Antiphospholipid antibodies may, therefore, play a diagnostic role for hereditary thrombophilia, as evidenced by the finding of these antibodies in a sizable portion of affected patients.

Elevated cytokine levels and immune dysregulation

Interleukin-6 (IL-6) and tumor necrosis factor-alpha (TNF-α) levels were higher in the study's thrombophilia group compared to the control group (p=0.029 and p=0.012, respectively), which is another important finding. These results are consistent with mounting evidence that immune dysregulation, characterized by an imbalance in cytokine profiles, is a key factor in repeated pregnancy loss [21,22]. Pro-inflammatory cytokines are known to disrupt the delicate immunological balance required to maintain pregnancy, which has been linked to adverse pregnancy outcomes, including recurrent miscarriages [23-25].

Hereditary thrombophilia and elevated cytokine levels together highlight the complex immune response in these patients. Hereditary thrombophilia-related thrombotic events may set off inflammatory responses, amplifying the pro-inflammatory milieu in the placental microenvironment. More research is necessary to understand the clinical implications and potential therapeutic interventions of this interaction between thrombotic and immunological processes.

Natural killer (NK) cells and pregnancy outcomes

Although the NK cell counts between the control and thrombophilia groups were not statistically different in this study (p=0.213), it is significant to note that NK cell activity and subsets may have a complex role in repeated pregnancy loss. Uterine NK cells aid in placental development during pregnancy, whereas peripheral NK cells have the potential to become cytotoxic when their regulation is off [26,27]. To better understand how NK cell subsets contribute to recurrent pregnancy loss in hereditary thrombophilia, future research should investigate these subsets' functional activities.

Clinical implications

This study's conclusions have a number of clinical ramifications. First, the importance of testing for antiphospholipid antibodies in people with a history of recurrent miscarriage is highlighted by the discovery of these antibodies in patients with hereditary thrombophilia. Antiphospholipid antibodies may present a therapeutic opportunity to enhance pregnancy outcomes in this population if they are identified early and managed. Second, the increased IL-6 and TNF-α levels in the thrombophilia group point to a potential role for immunomodulatory interventions in treating these patients' repeated pregnancy losses. As possible therapeutic approaches, reducing inflammation and reestablishing immune balance could be investigated. To determine the effectiveness and safety of such interventions, more research is necessary.

Study limitations

The study has several limitations, including a small sample size that may limit the generalizability of results, a cross-sectional design that makes it difficult to establish causality, and a focus on particular immunological markers rather than an entire immune profile that may have missed other factors influencing recurrent pregnancy loss in patients with hereditary thrombophilia.

Future research recommendations

Future studies should focus on thorough genetic and immunological profiling, carry out longitudinal studies with larger cohorts, and investigate the security and effectiveness of immunomodulatory interventions. Investigating particular NK cell subsets and their roles in recurrent pregnancy loss brought on by hereditary thrombophilia is crucial. Collaborations between experts from different fields can improve our comprehension of the intricate interactions between genetics and immunology, resulting in more precise diagnostic and therapeutic methods.

## Conclusions

In conclusion, this study has shed important light on the immunological risk factors connected to recurrent miscarriages in people with hereditary thrombophilia. The findings suggest that the pathogenesis of recurrent pregnancy loss in this population may involve complex interactions involving NK cells, elevated levels of pro-inflammatory cytokines (IL-6 and TNF-α), and antiphospholipid antibodies. These results underline the necessity of thorough screening and evaluation in women with hereditary thrombophilia who have repeatedly lost pregnancies. Monitoring cytokine levels and early antiphospholipid antibody detection may present opportunities for targeted interventions to enhance pregnancy outcomes. Further investigation into the mechanisms underlying these immunological factors and their clinical implications is also necessary.

Interdisciplinary collaborations between clinicians, geneticists, and immunologists are essential in the effort to address recurrent pregnancy loss. Such partnerships can aid in a more thorough comprehension of the complex interactions between genetic mutations and immunological elements, ultimately resulting in the development of more accurate diagnostic techniques and targeted therapeutic approaches. We can offer hope and better outcomes to couples struggling with the difficulties of recurrent pregnancy loss in the context of hereditary thrombophilia by expanding our knowledge in this area.
